# Chelation in Antibacterial Drugs: From Nitroxoline to Cefiderocol and Beyond

**DOI:** 10.3390/antibiotics11081105

**Published:** 2022-08-15

**Authors:** Davorka Repac Antić, Marijo Parčina, Ivana Gobin, Mirna Petković Didović

**Affiliations:** 1 Department of Microbiology and Parasitology, Faculty of Medicine, University of Rijeka, 51000 Rijeka, Croatia; 2 Department of Clinical Microbiology, Clinical Hospital Center Rijeka, 51000 Rijeka, Croatia; 3 Institute of Medical Microbiology, Immunology and Parasitology, Bonn University Hospital, 53127 Bonn, Germany; 4 Department of Medical Chemistry, Biochemistry and Clinical Chemistry, Faculty of Medicine, University of Rijeka, 51000 Rijeka, Croatia

**Keywords:** chelation, antibacterial drugs, nitroxoline, antibacterial modes of action (MOAs)

## Abstract

In the era of escalating antimicrobial resistance, the need for antibacterial drugs with novel or improved modes of action (MOAs) is a health concern of utmost importance. Adding or improving the chelating abilities of existing drugs or finding new, nature-inspired chelating agents seems to be one of the major ways to ensure progress. This review article provides insight into the modes of action of antibacterial agents, class by class, through the perspective of chelation. We covered a wide scope of antibacterials, from a century-old quintessential chelating agent nitroxoline, currently unearthed due to its newly discovered anticancer and antibiofilm activities, over the commonly used antibacterial classes, to new cephalosporin cefiderocol and a potential future class of tetramates. We show the impressive spectrum of roles that chelation plays in antibacterial MOAs. This, by itself, demonstrates the importance of understanding the fundamental chemistry behind such complex processes.

## 1. Introduction

The significance of antibacterial drugs in modern medicine can hardly be overstated. Since their discovery over 100 years ago, antibiotics (antibacterial drugs produced by living organisms) have prolonged the average human lifespan by 23 years [1], not just by treating infectious diseases, but also through their role in cancer treatment, transplant procedures, preventing biomaterial implant failure, and as immunosuppressants. Their mode of action (MOA), depending on the class, is commonly described as the inhibition of cell wall/enzyme/nucleic acid synthesis or metabolic pathways, or as interference with cell wall integrity [1,2,3]. With such descriptions, it is not surprising that chelation—a ubiquitous process in bacterial pathophysiology, and evident in humans through a plethora of metalloproteins—is easily overlooked as an important part of antibacterial MOAs. Indeed, the chelation process is vital in the MOAs of many classes of antibacterial drugs, in the strategies for fighting the alarming increase of microbial resistance, and in the mechanisms of microbial resistance itself.

The term “chelate”, derived from a Greek word for crab claw, designates a subgroup of complex (or coordination) compounds that contain polydentate ligands. Polydentate ligands (from *poly* meaning “many”, *dent* meaning “teeth”, and *ligare* meaning “to bind”) are molecules or molecular ions that surround a central metal cation (or rarely metal atom) and attach to it with at least two coordinate covalent bonds, forming a chelate. Typical bidentate ligands are ethylenediamine and the oxalate ion, while by far the most famous polydentate ligand is hexadentate EDTA (ethylenediaminetetraacetic acid). Polydentate ligands are often called chelating agents or chelators. Numerous biological molecules are chelates (e.g., hemoglobin, hemocyanin, vitamin B12, and other metalloproteins; chlorophyll), and numerous others can act as chelators (e.g., proteins, polysaccharides, and polynucleic acids). Note that generally, chelators have a strong tendency to chelate metal cations, and thus chelates represent a very stable type of compound.

In medicine, chelation is commonly associated with chelation therapy, which designates metal sequestration and elimination from the organism by transforming toxic metal complexes into new, non-toxic chelates that can be easily excreted [4]. Chelation therapy began during World War II when chemists at the University of Oxford searched for an antidote for Lewisite, an arsenic-based chemical weapon. The antidote, trivially named British anti-Lewisite or BAL, is a chelating agent. Chemically, it is an organic dithiol compound called dimercaprol. It was soon established that EDTA is a very effective chelator for treating lead poisoning, while deferoxamine—as an iron chelator—is given intravenously to treat iron poisoning. Chelation therapy is also used to treat copper overload disorder such as in Wilson’s disease. There are attempts to implement “chelation therapy” in curing other kinds of diseases, such as cardiovascular disease, cancer, autism, and dementia [5,6,7,8]. Penicillamine, 8-hydroxyquinolines, deferiprone, and deferoxamine are also used clinically for chronic diseases. 

After the golden era of antibiotic discovery, from the 1940s to the 1960s, came the period characterized by scarce new discoveries, which continues today. Most of the then-discovered antibiotics are still in clinical use, but a dramatic rise in antimicrobial resistance (AMR) has rendered them less effective. Indeed, AMR is recognized as one of the most serious global public health threats of this century [9]. This has led to a revival of research regarding antibiotics, including the discovery of new ones, developing the existing ones using new tools and techniques, or re-examining and repurposing the old ones. The example depicting the latter case is nitroxoline [10,11,12,13,14,15,16,17,18,19], one of the oldest antibiotics, which does not belong to any of the typical antibacterial classes [20,21]. Chemically, it is a derivative of 8-hydroxyquinoline, and as such it belongs to a group of molecules with a so-called “privileged structure”, giving it a rich diversity of biological properties [22]. It is arguably the only antibiotic in use whose MOA rests purely on the chelation process. Therefore, in this review, we pay particular attention to nitroxoline and its MOA. 

The most obvious connection between chelation and antimicrobial activity, as reflected in nitroxoline’s MOA, lies in the fact that (transition) metal cations are essential for bacterial survival [5]. It has been known for a long time that microbial pathogens must acquire nutrient metals in order to cause disease. To secure iron ions, they produce siderophores, small organic molecules with various chemical structures, but always with the ability to strongly chelate Fe^3+^ [23]. Once caught by a siderophore, the Fe^3+^ ion is “trapped” in a stable chelate, which enters the microbial cell by an active transport mechanism. Then, Fe^3+^ is reduced to Fe^2+^, which has a diminished affinity for siderophore ligands, and is thus released from the chelate inside the cell. Novel cephalosporin antibiotic cefiderocol owes its potent activity to the ability to act as a siderophore [24,25]. Chelating agents are thus able to control in vivo infection progression by selectively disturbing the essential metal metabolism of the microorganism, such as the blockage of microbial nutrition, development and growth, or by disturbing the adhesion to biotic and abiotic surfaces [26,27,28,29,30]. Besides this recognizable employment of chelation, there are numerous other, more intricate applications in antimicrobial activities of other drugs. 

In this review, we explore and present the ways that chelation is involved in MOAs of a wide scope of antibacterial drugs, class by class, from a century-old nitroxoline to new cefiderocol and potentially new antibiotics tetramates. We also convey the role of chelation in the mechanisms of microbial resistance and in the development of new or improved antibacterials. First, we present a short overview of chelation activities (Table 1), which is followed by more elaborate descriptions, explanations, and schematic representations.

## 2. Class-by-Class Overview of Chelation Relevance

**Table 1:** An overview of the chelation relevance in modes of actions (MOAs) of different antibacterial drug classes.

Antibacterial CLASS/DRUG	References
Nitroxoline	
Antibacterial MOA rests entirely on its chelation ability Chelates divalent cations (Mn^2+^, Mg^2+^, Zn^2+^), with exception of Ca^2+^ Acts bacteriostatically by chelating cations essential for bacterial growth Chelation also determines its antifungal, anticancer, neuroprotective, antischistosomal and antileishmanial activities, and bioactivity in general	[11,18,19,21,31,32,33,34,35]
Tetracyclines	
Ability to chelate metal ions is crucial for their pharmacological activity Able to chelate divalent and trivalent metal cations A tetracycline molecule is able to inhibit bacterial growth only in the form of an Mg-chelate Transported in the blood plasma as for the Ca-chelates Several coordinating sites, extraordinary coordination ability Chelation also governs the mechanism of bacterial resistance to tetracyclines	[36,37,38,39,40,41,42,43]
Fluoroquinolones	
The core of their MOA is the chelation of non-catalytic Mg^2+^ cation of bacterium topoisomerase IV Powerful iron chelators comparable with deferoxamine Able to act as monodentate, bidentate, or a bridging ligand	[44,45,46]
Sulfonamides	
They do possess chelating abilities, but their antibacterial MOA does not include chelation Newer studies indicate increased antibacterial and antifungal activities when in a form of a chelate New generation derivatives show antibacterial and antioxidative activities as 2:1 chelates	[47,48,49]
Polypeptide Antibacterials	
Vancomycin: MOA of unmodified vancomycin does not include chelation, but the loss of efficiency due to bacterial resistance can be circumvented by the addition of the Zn-chelating moieties to basic vancomycin structure; Cu-chelates show improved enantioselectivity Polymyxin: MOA does not include chelation by the polymyxin itself; instead, it is based on the disruption of native Mg- and Ca-lipopolysaccharide chelates in Gram-negative bacteria membrane Bacitracin: MOA includes the chelation of divalent metal ions like Zn^2+^ in order to increase adsorption of bacitracin to bacterial cell surface; Zn^2+^ chelate serves as a connection between bacitracin and a membrane pyrophosphate, resulting in the inhibition of cell-wall biosynthesis	[50,51,52,53,54,55,56,57,58,59,60,61,62,63]
Macrolides and Lincosamides	
MOA relies on blocking the protein synthesis by binding to ribosomal 50S subunit, in a manner that does not include chelation	
Cefiderocol and Other Beta-Lactam Antibacterials	
Cefiderocol is intentionally designed as an “upgrade” of existing β-lactam antibiotics to improve the chelating abilities, by the addition of an iron-chelating chlorocatechol moiety Cefiderocol-Fe^3+^ chelate uses a bacterial ferric ion transport system (the Trojan horse approach) to overcome the decreased permeability of Gram-negative bacteria membrane MOAs of other β-lactam antibiotics do not include chelation β-lactamase class B are metalloenzymes, helping bacteria destroy the β-lactam antibiotic by chelating the antibiotic’s β-lactam ring using zinc or other metal cations Zinc chelators are considered potential carbapenem adjuvant molecules (restoring carbapenem activity) against metallo-β-lactamase	[24,25,27,64,65,66,67,68,69,70,71]
Tetramates	
Potential new antibacterial class, discovered during the search for new substances that could mimic natural products with chelating properties The ability to reduce Fe^3+^ to Fe^2+^ after chelation triggers Fenton reactions (oxidative attack by the production of deleterious hydroxyl radicals), resulting in bacterial cell death Similarly to nitroxoline, their antibacterial actions are directly correlated to their chelating capabilities	[72,73,74]

## 3. Nitroxoline

Nitroxoline (5-nitro-8-hydroxyquinoline, NIT) is a heterocyclic aromatic compound from a family of 8-hydroxyquinoline (8-HQ, or oxine) derivatives, a group of compounds with an extremely broad spectrum of pharmacological [16,20,22,35,75,76] and non-pharmacological [77,78,79] applications. NIT does not belong to any typical class of antibacterials, and it is best known as an effective uroantiseptic that shows no pronounced toxic side effects [16,19,80,81]. Its use dates back to the beginning of the 20th century [82,83], but is currently becoming an option for the treatment of infections caused by multidrug-resistant uropathogens, primarily due to an increase of the bacterial resistance to more commonly used antibacterials and its unique MOA [10,13,20]. Out of all classes of antibacterials, 8-HQ derivatives are the only ones currently in use whose antibacterial actions, and bioactivity in general, rest almost exclusively on their chelation properties [31,35,79,82,84]. Chelation is also responsible for NIT’s unique ability to inhibit the formation of bacterial biofilm (i.e., biofilm activity) [12,18], as explained in the following paragraph. 

**Figure 1:**
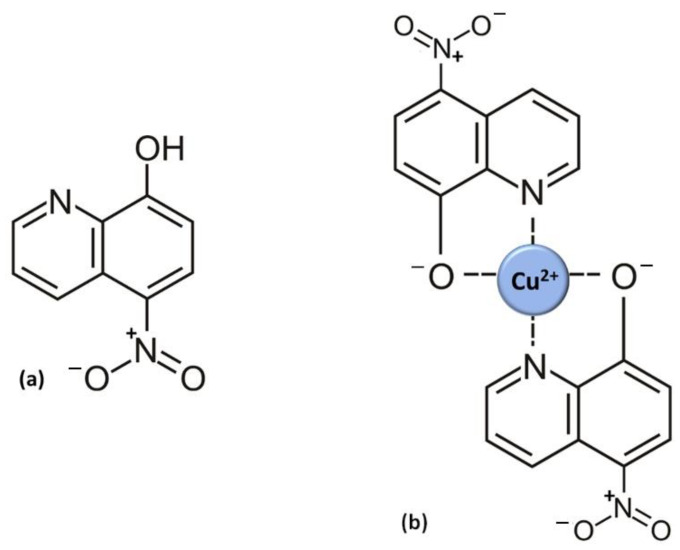
(**a**) Structure of nitroxoline (5-nitro-8-hydroxyquinoline, NIT); (**b**) example of NIT−Cu^2+^ chelate, showing two possible chelating sites.

The 8-HQ core has a so-called “privileged structure”, a concept designating molecular scaffolds with versatile binding properties [85]. The core structure is small and simple, only consisting of a phenol and a pyridine ring, with a hydroxy group in close proximity to pyrimidine’s nitrogen (Figure 1a). The acidic properties of the hydroxy group are vital: p*K*a of the –OH group is 6.3 [21], signifying that at pH = 6.3, half of the hydroxyls are deprotonated; complete deprotonation is achieved at pH = p*K*a + 2. Chelation is proportional to the number of deprotonated hydroxyls; hence, it is more pronounced at higher pH values. This is due to the fact that the phenolate oxygen (–O^−^) is the atom that chelates metallic cations, i.e., the first chelating position. The second chelating position is the nitrogen of the pyrimidine ring, as shown in Figure 1b. This figure also shows 2:1 stoichiometry (two ligands per one metal cation), which was shown to be exact for all other 8-HQ derivatives as well [21]. The presence and the position of the nitro group, –NO_2_, were shown to be crucial for nitroxoline MOA: none of the other 8-HQ derivatives demonstrate such antibacterial actions [15,35]. The p-position of –NO_2_ reinforces hydroxide group acidity, enhancing the chelating ability for the abovementioned reasons [86]. Furthermore, it acts as a nitrogen radical source that alters intracellular signaling, leading to the inhibition of tumor cell growth [79,80], and provides a point of interaction with two histidines of human cathepsin B, enabling its inhibition [19,87].

**Figure 2:**
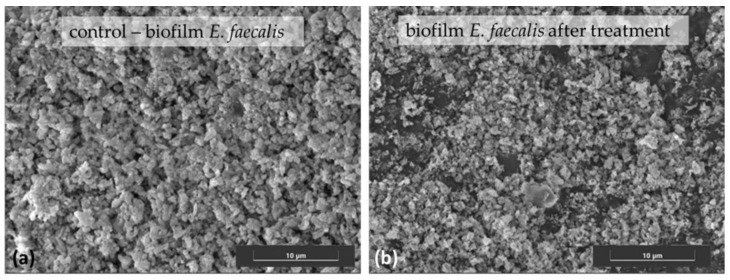
SEM images of *E. faecalis* on urinary catheter surface (**a**) before and (**b**) after nitroxoline treatment, showing the areas of biofilm destruction. Samples were coated with Pd before analysis on a Hitachi S–3600N Scanning Electron Microscope. Image taken at 13.3 k magnification (scale bar represents 10 μm).

As aforesaid, it is known that NIT’s antibacterial activity is due to its chelating ability, although the details of its antibacterial MOA are still not elucidated. Generally, it is considered that its activity is indirect: NIT acts bacteriostatically by chelating cations essential for bacterial growth. Pelletier et al. [21] found that nitroxoline’s bacteriostatic and bactericidal activities were reduced by the presence of Mg^2+^ and Mn^2+^, while K^+^, Na^+^, and Ca^2+^ did not exhibit any influence. The premise is that NIT interacts with Mg^2+^ and Mn^2+^, thus the chelating sites become presaturated in their presence, rendering NIT unable to chelate divalent cation within the bacterial cell, proving that chelation is the essence of NIT’s MOA. Mg^2+^ was more efficient in reducing NIT activity compared to Mn^2+^, which was attributed to a stronger stabilization of the outer membrane with high external Mg^2+^ concentration, which limited the diffusion through the membrane. The authors suggest a possible similarity to the MOA of 8-HQ in yeast [32] and *E. coli* [33], where 8-HQ inhibits RNA polymerase solely by chelating the Mg^2+^ and Mn^2+^ required for its activity, without any direct contact between 8-HQ and the enzyme. However, this does not exclude the possibility of alternative MOE in vivo [33]. Sobke et al. [18] found that NIT antibiofilm activity is indirect as well, i.e., mediated by the removal of free iron and zinc cations. The authors speculate that the chelation of iron and zinc cations disabled their role in regulating type IV pilus expression, resulting in decreased biofilm formation. On the other hand, Latrache et al. [34] showed that NIT did not inhibit the expression of fimbriae. The same study also showed that bacterial cell surface hydrophobicity increased in the presence of NIT, but—contrary to expectations—the adhesion to a hydrophobic catheter surface decreased, clearly demonstrating the well-known complexity of the bacterial adhesion process [88]. In an attempt to further elucidate the mechanism of NIT influence on bacterial adhesion, we studied the adhesion of *E*. *faecalis*, a known uropathogen, on a catheter surface. Our preliminary results showed that NIT indeed inhibited *E. faecalis* adhesion and demonstrated antibiofilm action (Figure 2) as well.

More recent studies focused on methionine aminopeptidases (MetAP), a class of metalloenzymes present both in humans and microorganisms, as a molecular target for 8-HQ derivatives [35]. Among them, NIT showed pronounced results in the inhibition of MetAP from *Burkholderia pseudomallei*, bacteria resistant to several antibacterial drug classes that cause critical infection. The ability to chelate (or coordinate, which would be accurate terminology in this case) was again the core of NIT’s MOA: pyridine nitrogen and phenolate oxygen coordinate two metal cations in enzyme active site, thus inhibiting its activities. The inhibition of these enzymes in bacteria leads to impaired cell growth and death; in humans, it leads to antiangiogenic activity. The antiangiogenic and anticancer activities of NIT were also proven in vivo, in a study that showed the statistically significant inhibition of angiogenesis and tumor growth in the breast cancer xenograft model [89].

## 4. Tetracyclines

Discovered as natural products from actinomycetes soil bacteria, tetracyclines (TCs) were first reported in the scientific literature in 1948 [90]. For all members of this pleiotropic class of drugs, the ability to chelate divalent and trivalent metal cations dictates their pharmacological activity, and they are known as strong chelating agents [38,91].

**Figure 3:**
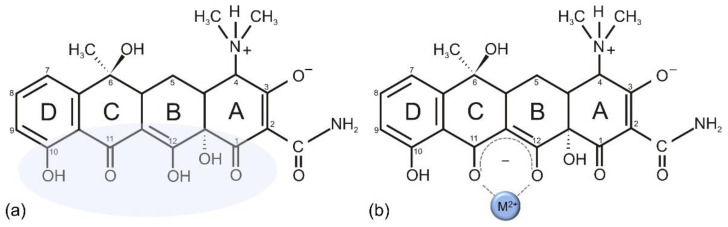
(**a**) Structure of tetracycline. The shaded area emphasizes the part relevant for chelating ability. (**b**) An example of a simple tetracycline–Mg chelate.

TCs’ molecular structure is quite complex, consisting of four hydrocarbon rings bearing numerous functional groups capable of chelation (Figure 3a). Hydroxyl groups and a dimethylammonium group located at C4 can be deprotonated; thus, the overall charge of the molecule is pH-dependent, which was proven to be crucial for their MOA. At physiological pH, the molecule assumes the zwitterionic form. Along with pH, the polarity of the solvent is also relevant and determines which of the multiple conformations the molecule will assume. Such diversity makes it difficult to accentuate a single representative TC chelate: various chelates differing in metal-to-ligand stoichiometry and chelation modes have been proposed [38]. However, the lower side of the molecule (shaded area in Figure 3a) is recognized as relevant for metal chelation due to the abundance of oxygen atoms; the example of a basic chelate is shown in Figure 3b [40,92]. The dimethylammonium group, located at C4, was shown to be vital for TC antibacterial activities because it enables the formation of the zwitterionic form. Generally, the part of the surface from C1 to C4 and C10 to C12 is hydrophilic, while the rest (C5 to C9) is hydrophobic [42]. 

**Figure 4:**
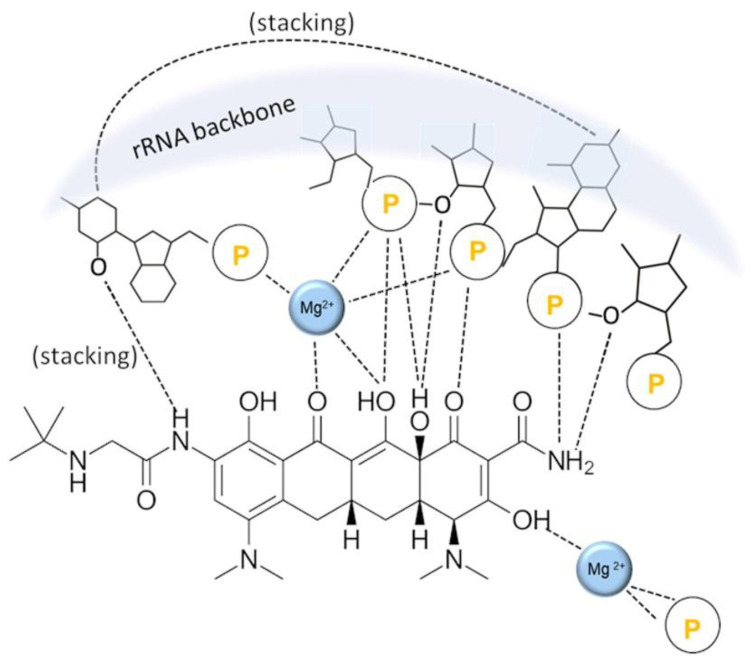
Binding of the TC molecule (tigecycline) to phosphate group (P) oxygens of the rRNA backbone in the bacterial 30S ribosomal subunit. The binding is done both directly and via Mg^2+^ ion(s). The image is inspired by ref. [93].

The role of chelation in the MOA of TCs is intricate and fascinating [37,39,92]. In order to diffuse through the bacterial cell membrane, a TC molecule has to be fully protonated; the Mg-chelate cannot enter the cell. Which of the two will be a dominant form is determined by pH and Mg^2+^ concentration: higher pH and higher Mg^2+^ concentrations favor the Mg-chelate form [37,39]. Both pH and Mg^2+^ concentrations are higher inside the bacterial cell, and this pH difference is the driving force of the TC uptake (transport proteins are obsolete) [39]. Hence, when the fully protonated TC enters the bacterial cell, it encounters a milieu that shifts the equilibrium towards Mg-chelate, which is not able to escape the cell. It has been shown that only in the form of an Mg-chelate is a TC molecule able to inhibit bacterial growth, by binding to the bacterial 30S ribosomal subunit. In particular, the TC molecule binds with its hydrophilic side to phosphate group oxygens of the rRNA backbone both directly and via Mg^2+^ ion(s) [42,93,94,95], as shown by the example of tigecycline in Figure 4. 

Chelation also governs the mechanism of bacterial resistance to TCs [37,41]. Moreover, it is also vital for the TC actions in other, non-microbial diseases. It has been shown that the binding of TCs to proteins, such as matrix metalloproteinases, is greatly enhanced if the TC is in the form of a chelate with Ca^2+^ or Mg^2+^ [96] and can be mediated by the chelation of Zn^2+^ ions [97,98].

It is well known that antacids and dairy products impair the efficiency of TCs, which is a direct consequence of their strong chelating ability. They form insoluble or poorly soluble chelates with cations contained in those products, which interferes with TC absorption from the gastrointestinal tract by 50% to 90% [99,100]. On the other hand, several studies have demonstrated the chelator-based protective mechanism, i.e., the benefit of using iron chelators in conjunction with most tetracyclines [101,102]. By chelating iron instead of the TCs, iron chelators preserve the binding of tetracyclines to the bacterial ribosome and, hence, their antimicrobial activity.

The accumulation of TCs and other antibiotics in the environment poses a serious threat to human health [103]. The removal of TCs from the aqueous environment is possible again by exploiting their chelating ability. For example, it was shown that the adsorption capacity of mesoporous silica for TCs was improved by grafting the silica surface with amino groups and Fe^3+^. The TCs adsorbed by forming a complex with Fe^3+^ through the nitrogen from the dimethylammonium group located at C4 (Figure 3) [104].

## 5. Fluoroquinolones

Fluoroquinolones (FQ) were discovered in the 1960s as a derivative of the antimalarial drug chloroquine, and they have been widely used in human and veterinary medicine since the late 1980s. In this class, chelation is also at the core of their MOA.

**Figure 5:**
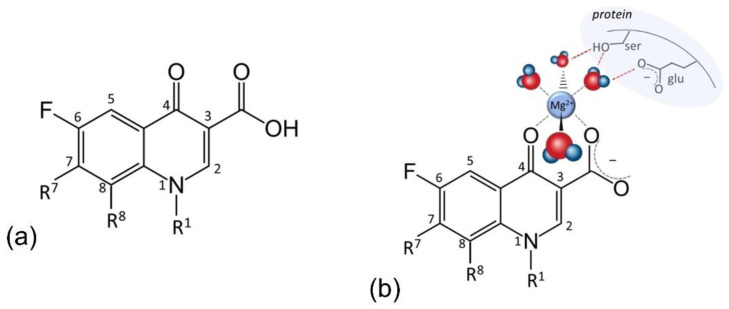
(**a**) General structure of fluoroquinolones. (**b**) Chelation of Mg^2+^ by a fluoroquinolone molecule (moxifloxacin), as the core of their mode of action. Two water molecules serve as a bridge to bacterium topoisomerase IV (hydrogen bonds indicated by red dashed line), blocking phosphotyrosine from approaching the other Mg^2+^ at the enzyme’s active site. Hydrogen bonds with DNA strand also present are not shown for simplicity. Inspired by ref. [45].

Quinolones in general are a group of synthetic antibacterial agents containing a heterocyclic 4-oxo-1,4-dihydroquinoline skeleton, with a carboxylic group at C3 and usually one other N-heterocyclic ring such as piperazinyl at C7 (Figure 5a). Fluoroquinolones are a subgroup containing many members, all of them comprising a fluorine atom at C6 and various smaller (cyclo)alkyl group at R1. Besides hydrogen, R8 can be a methoxy group or another halogen (fluorine or chlorine). Rarely, C8 is replaced by nitrogen. Such a structure makes them able to act as a monodentate, bidentate, or a bridging ligand, with multiple modes of chelation and coordination [105]. In their antibacterial activities, the main targets are gyrases (type II topoisomerases) and topoisomerase IV, enzymes that participate in DNA replication [106]. It has been known for a long time that magnesium ions play a crucial role in their MOA [44], but final elucidation was given in studies showing that FQ (moxifloxacin) is able to chelate the non-catalytic Mg^2+^ cation of bacterium topoisomerase IV through the oxygens of the C4 keto and C3 carboxyl group (Figure 5b) [45,46]. Alongside the oxygen atoms, the Mg^2+^ ion is coordinated to four water molecules. Two of them serve as a bridge to the enzyme by forming hydrogen bonds with a nearby serine and glutamic acid residue. Other water molecules are coordinated to oxygen atoms from a DNA strand. In this manner, the FQ molecule blocks the phosphotyrosine from approaching the active site (catalytic) Mg^2+^ ion. 

FQ are also powerful iron chelators comparable with deferoxamine. Only when given orally, drug–drug interactions due to chelation can occur between fluoroquinolones and divalent or trivalent cation-containing compounds such as iron, calcium, zinc, magnesium, and aluminum. This chelation leads to a formation of an insoluble complex compound that is poorly absorbed from the gastrointestinal tract. Co-administration at the same time and inappropriate separation may lead to a clinically significant decrease in antibiotic bioavailability, resulting in an increase in bacterial resistance and treatment failure [43]. On the other hand, it has been proven in many ways that the metal complexes of drugs are more active than their parent compounds [107]. Diverse metal complexes with quinolones have been synthesized in an attempt to investigate the physicochemical properties and to evaluate their antibacterial activity in comparison to free quinolone [108,109]. The synthesis and characterization of zinc complexes with the quinolone antibacterial drugs have been achieved. The complexes exhibit significant antibacterial activity against a few Gram-positive and Gram-negative bacterial strains, which is higher than the corresponding free quinolone antibacterial drugs [106].

## 6. Sulfonamides

The use of sulfonamides as antimicrobial agents is a consequence of the discovery (by Gerhard Domagk, for which he won the Nobel Prize in medicine in 1939) that dyes can be used as antibiotics. They are the first successfully synthesized antibacterial drugs.

**Figure 6:**
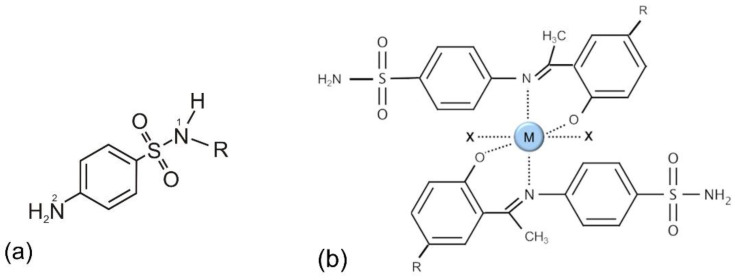
(**a**) General structure of sulfonamides. (**b**) Chelation is not relevant in the classical sulfonamide mode of action, but the chelates of new-generation sulfonamide molecules with metal (M) cations (M = Co^2+^, Ni^2+^, Cu^2+^, Zn^2+^, and VO^2+^) demonstrate antibacterial and antioxidative activities; inspired by ref. [49].

The core of their structure consists of a benzene ring with a directly attached sulfonamide group and an amine group in para-positions (Figure 6a). One hydrogen on sulfonamide nitrogen (N1) can be substituted by various groups (usually some N-heterocyclic group), yielding numerous members of this group of antibacterials. Rarely, the substituents are added on N2. The presence of additional groups in ortho- and meta-positions diminishes their antibacterial activity, as well as the double substitution on N1, due to the fact that their MOA is based on mimicking p-aminobenzoic acid (PABA). The structural similarity tricks the enzyme (dihydropteroate synthase) to bind sulfonamide instead of PABA, and consequently, folic acid and DNA synthesis in the bacteria are prevented [110]. This MOA is efficient since bacteria are obligate folic acid synthesizers, while humans obtain folate through dietary sources [111]. This type of MOA does not include any chelation.

However, sulfonamides do have chelating abilities, which have been known of and used since the 1960s in analytical chemistry, namely, metal analysis and separations [112]. The survey of the literature since then reveals that sulfonamide-based Schiff bases and metal complexes are imperative due to their biotic activities. These ligands and their metal chelates were shown to have antioxidant- and enzyme-inhibiting abilities. Antibacterial and antifungal screenings have shown that the chelates were more efficient in that sense than the free ligands [49]. Furthermore, the studies have also shown that multi-drug resistance may be combated in the future via metal-based medicines derived from several commonly used pharmaceuticals such as sulfonamides [49,113]. One study presented the synthesis of a new derivative of N-carboxamide compound bearing sulfonamide group and its Zn^2+^, Ni^2+^, Mn^2+^, Cu^2+^, Co^2+^, and Pd^2+^ complexes [114]. Mn and Pd complexes showed potential inhibition activities against the tested microorganisms. In one report, new sulfonamide-based ligands were synthesized, and the results indicated that the complexes may be considered for further drug design endeavors [115]. In 2006, a series of copper complexes of heterocyclic sulfonamides with antibacterial activity was reported [116]. More recently, sulfonamides and their metal-based (cobalt, copper, nickel, or zinc) compounds were synthesized and screened for in vitro antibacterial and antiparasitic activity [47,48]. Finally, in 2022, bidentate chelates of sulfonamide and metals (Co^2+^, Ni^2+^, Cu^2+^, Zn^2+^, and VO^2+^) were synthesized and screened (Figure 6b), and some of these demonstrated antibacterial and antioxidative activities [49].

## 7. Polypeptide Antibacterials

### 7.1. Vancomycin

Vancomycin, a representative of the glycopeptide group of antibiotics, is a product of the bacterium *Amycolaptosis orientalis*. This antibiotic was discovered in 1956 in the fermentation filtrate of this bacterium. It has a wide range of action on bacteria, but its effectiveness is increased against Gram-positive bacteria.

Antibiotics of the vancomycin group bind peptides in bacterial cell wall precursors, which prevents the precursors from crosslinking adjacent strands, consequently weakening the cell wall integrity [53]. The binding does not include chelation [117].

**Figure 7:**
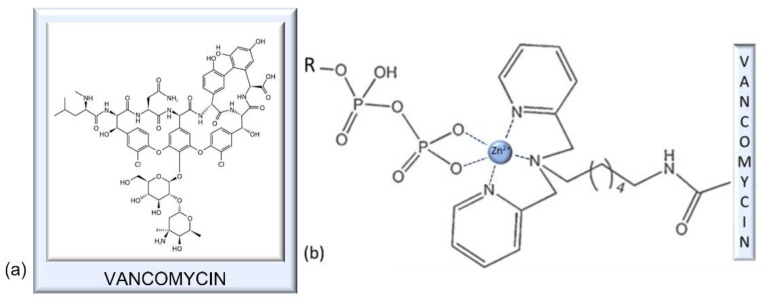
(**a**) Structure of vancomycin, a glycopeptide antibiotic. (**b**) Zinc chelate of dipicolyl–vancomycin conjugate and pyrophosphate groups of cell-wall lipids. Inspired by ref. [50].

Vancomycin has since lost much of its efficacy due to the emergence of vancomycin-resistant bacteria (VRB), such as vancomycin-resistant *S*. *aureus* (VRSA), vancomycin-intermediate-resistant *S*. *aureus* (VISA), and vancomycin-resistant Enterococcus (VRE). One of the ways to combat this threat is to modify the basic structure using metal-chelating, lipophilic, and galactose-attachment strategies [51]. The efficiency of the metal-chelating strategy was demonstrated in a study where a dipicolyl moiety—a zinc-binding ligand—was attached to vancomycin (Figure 7b) [50]. The dipicolyl moiety chelated Zn^2+^ from three positions, while the two other positions were occupied by oxygen atoms from pyrophosphate of the cell-wall lipid, which resulted in the enhanced inhibition of bacterial cell-wall biosynthesis. This modification yielded a 375-fold increase in effectiveness against one phenotype of VRB and, furthermore, did not induce the development of bacterial resistance. Another study indicated that vancomycin itself can act as a zinc chelator, and as such, can induce a zinc starvation response in treated bacteria [118].

The chelation of other metal cations also proved to be an efficient strategy in improving vancomycin’s antibacterial actions. For example, the effect of the iron chelator deferasirox on vancomycin’s efficacy against two methicillin-resistant *S*. *aureus* (MRSA) strains was examined in vitro and in a murine bacteremia model [119]. The results showed that deferasirox treatment significantly enhanced the capacity of vancomycin to bind to the cell surface of *S*. *aureus*; thus, iron chelation was considered to be a promising, novel adjunctive therapeutic strategy for MRSA and VISA infections. Moreover, it was shown that vancomycin can chelate Cu^2+^ ions by acting as a tetradentate ligand, which improved its enantioselectivity [52].

### 7.2. Polymyxins 

Polymyxins are the products of different species of the genus *Bacillus* and are currently used as a last resort defense against difficult multidrug-resistant pathogens. They were discovered as early as 1947. In 1948, a Washington newspaper wrote “Time will tell, however, whether polymyxin will work in human beings” [120]. Indeed, time told a very dramatic story: at first, they were considered “miracle” antibiotics, but soon they were infamous due to nephrotoxicity. In modern times, similarly to nitroxoline, they have experienced a renewed clinical interest due to rising bacterial resistance [56,57,121].

**Figure 8:**
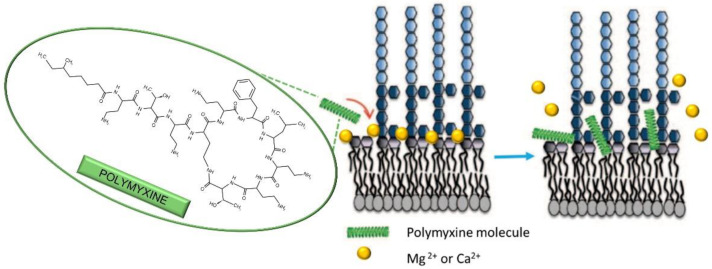
One of the proposed modes of action of polymyxins on Gram-negative bacteria. Due to higher affinity to membrane lipopolysaccharides (LPS) compared to native Mg^2+^ and Ca^2+^, polymyxine molecules displace the cations from LPS-cation chelates (crucial for membrane stability), thus disrupting the physical integrity of the membrane. Inspired by ref. [55].

Surprisingly, polymyxins’ MOA is still a subject of debate [56], but generally, it is accepted that the bacterial membrane is the primary target. The Gram-negative bacterial membrane comprises lipopolysaccharides (LPS), phospholipids, proteins, lipoproteins, etc. LPS are polyanionic molecules, and as a result, an accumulation of negative charges occurs in the lipid A and core oligosaccharide regions. The repulsive forces between negative charges are counterbalanced by the bridging Mg^2+^ and Ca^2+^ cations, and this chelation is crucial for the integrity of the bacterial outer membrane (Figure 8) [60,122]. One of the relevant models of MOA proposed that the first step in polymyxin’s destruction of the membrane is the displacement of Mg^2+^ and Ca^2+^ cations by the polymyxin molecule, which has at least three-fold higher affinity to LPS compared to native Mg^2+^ and Ca^2+^. The disruption of Mg^2+^/Ca^2+^-LPS chelates and the insertion of bulky polymyxin molecules instead results in the “crack” formation that allows for the passage of other molecules, among which are the other polymyxins. The insertion expands and weakens the membrane, finally resulting in the destruction of the physical integrity of the membrane [55,56,57,58,59]. Thus, in the case of polymyxins, chelation is not done by them, but the disruption of native chelates present in bacterial membranes represents the core of their MOA.

### 7.3. Bacitracin

**Figure 9:**
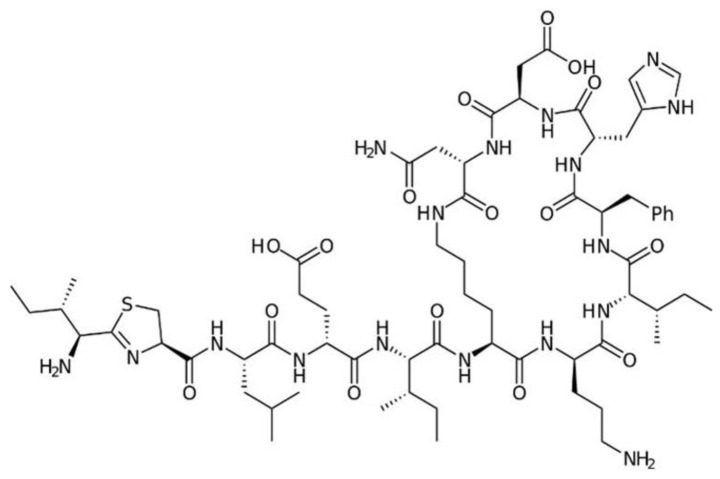
Structure of bacitracin.

Bacitracin (Figure 9) was discovered in 1945 as a product of the bacterium *Bacillus licheniphormis*. The antibacterial spectrum of bacitracin is Gram-positive and includes staphylococci, streptococci, Corynebacterium, and Clostridium, with rare resistance seen in staphylococci. Bacitracin is too toxic to be used parenterally, but is well tolerated topically.

Its antibacterial MOA is the inhibition of the cell wall synthesis [61], through the inhibition of peptidoglycan synthesis, where the chelation is a vital step. Bacitracin forms chelates with divalent metal ions like Zn^2+^ in order to increase the adsorption of bacitracin to the bacterial cell surface. Bacitracin requires the binding of divalent metal ions for sequestration of its molecular target undecaprenyl-pyrophosphate, with Zn^2+^ being the most effective in stabilizing complex formation through the neutralization of the pyrophosphate charge [62]. Zn^2+^ ion serves as a connection between bacitracin and a pyrophosphate, by being coordinated with two pyrophosphate oxygen atoms, and three donor atoms from bacitracin. The imidazole ring of histidine in bacitracin was proven crucial for the binding of metal ions to the diphosphate moiety [123]. By binding to pyrophosphate, bacitracin prevents the transport of peptidoglycan precursors through the membrane and thus disrupts cell wall biosynthesis.

Antimicrobial resistance has become a threatening health issue in recent years, but bacterial resistance toward bacitracin is still limited despite its wide use in the past several decades. Thus, it can serve as a potential lead for the design of potent antibiotic metallopeptides and analogs, with limited potential to evoke bacterial resistance for combating bacterial infection [63].

## 8. Macrolides and Lincosamides

**Figure 10:**
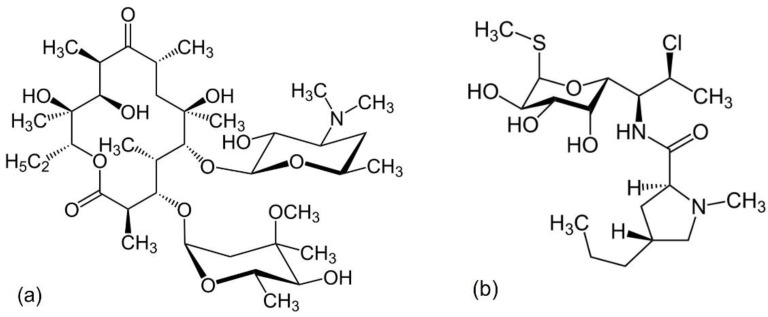
Generic structures of (**a**) macrolides and (**b**) lincosamides.

Erythromycin A, which was introduced into clinical practice in 1953, represented the first generation of macrolides (Figure 10a). The lincosamide class of antibacterials (Figure 10b) originates from a natural product, lincomycin, and alongside lincomycin, includes the semisynthetic derivatives clindamycin and pirlimycin. This class was first characterized in the 1960s and is now used for the treatment of a broad spectrum of infections [124]. Both classes of antibacterials block protein synthesis by interacting with the large ribosomal 50S subunit, with MOA that does not include chelation [29,125,126]. 

Co^2+^ complexes of lincomycin and its derivatives were investigated for other biological applications [127].

## 9. Cefiderocol and Other Beta-Lactam Antibacterials 

**Figure 11:**
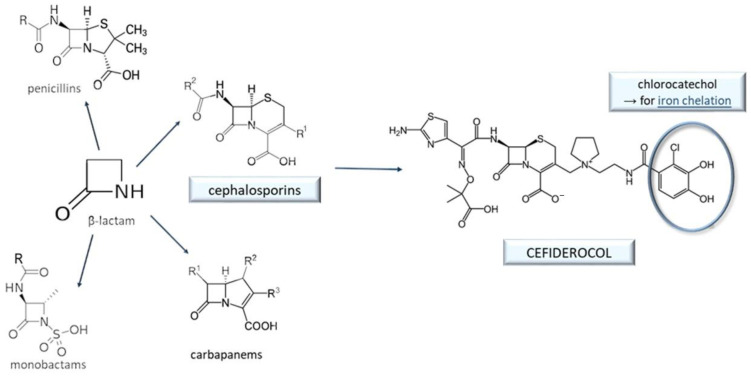
Classification of the β-lactam antibacterials. Cefiderocol, a member of the cephalosporin subgroup, contains a catechol moiety that enables it to chelate Fe^3+^ ions and thus use the bacterial ferric ion transport system to enter the bacterial cell (the Trojan horse approach).

Quite the opposite of nitroxoline—a chelating antibacterial drug in use for more than half a century, unearthed subsequently as a high potential anticancer agent—cefiderocol is a new drug [128], designed intentionally as an “upgrade” of existing β-lactam antibiotics to improve the chelating abilities. Inspired by natural siderophores from *E*. *coli* and *P*. *aeruginosa*, the catechol moiety is added at the end of one side chain (Figure 11) [64,65]. It serves as a co-called linker [27], connecting the antibiotic to Fe^3+^ ions and thus forming the chelate that is able to use the bacterial ferric ion transport system to overcome the decreased permeability of Gram-negative bacteria [24,129]. This “Trojan horse” approach is very efficient in circumventing certain intrinsic or acquired antibiotic resistance mechanisms [64,130]. Cefiderocol was successfully approved in 2019 for the treatment of complicated urinary tract infections and hospital-acquired bacterial pneumonia, and in 2020, for ventilator-associated bacterial pneumonia caused by GNB [25].

**Figure 12:**
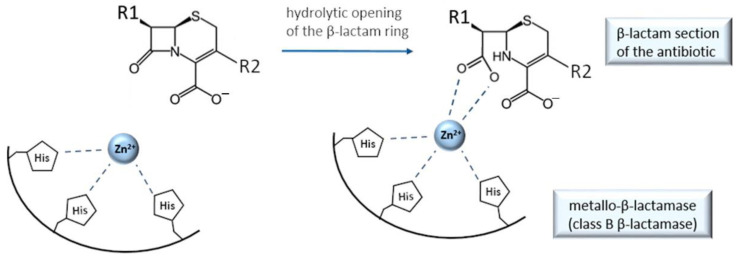
Simplified scheme of metallo-β-lactamase catalytic mechanism, depicting the role of chelation in disabling the action of β-lactam antibiotics (inspired by ref. [71]). For clarity, only one out of two Zn^2+^ ions are shown.

Other β-lactam antibacterials achieve their bactericidal activities by inhibiting an enzyme involved in bacterial cell wall synthesis. Concretely, they act as a “suicide substrate” to penicillin-binding proteins (PBPs) by covalently binding to PBPs serine residue [131]; thus, their MOA does not include chelation. They are one of the most successful classes of antibiotics, but their overuse brought about bacterial resistance on an alarming scale. Bacteria have developed multiple resistance strategies, one of which is the production of the enzymes β-lactamases. This family of enzymes hydrolyses the C–N bond within the β-lactam ring (Figure 12) and thus inactivates the antibiotic. There are four classes of β-lactamases (A, B, C, and D), among which class B lactamases are recognized as a particular clinical threat due to the fact that they cannot be inactivated by clinically useful inhibitors and are extremely promiscuous [71,132]. What distinguishes the class B lactamases from the other three classes is the fact class B uses chelation to destroy antibiotics’ β-lactam ring, while the classes A, C, and D employ covalent bonding through the active site serine. Class B are thus metallo-β-lactamases (MBLs), while classes A, C, and D are referred to as serine-β-lactamases [133]. Chelation in class B is done via (one or two) Zn^2+^ or other metal cations [70,71], as shown in a simplified manner in Figure 12.

The mechanism includes two Zn^2+^ ions and one water molecule that is activated by them to carry out the hydrolyses of the β-lactam ring [70]. That being said, it is easy to comprehend that MBLs could be inhibited by some species that can seize Zn^2+^ ions from them, i.e., some Zn^2+^ chelators. The main contesters are molecules containing chelating groups (thiols, carboxylates, etc.) combined with an aromatic group, and nitroxoline (Section 2) is one of them [69]. Those molecules could then be used as antibiotic adjuvants, and as such provide a last resort in fighting infections caused by MBL-producing bacteria. What is not easy, on the other hand, is to find Zn^2+^ chelators that would not be toxic to humans due to cross-reactivity with human metalloenzymes. Hence, this is an important topic of current public health research.

## 10. Outlook: Chelation in Current Antibacterial Research and New Directions

The emergence of multidrug-resistant bacterial infections is prompting the ongoing research efforts into finding new antibacterial agents, which has resulted in approval of 12 new antibacterial drugs since 2017 [134]. One of them is cefiderocol, described in detail in Section 9; three belong to flouroquinolones (Section 5); and the other two are tetracycline-based drugs (Section 4). Two new polymyxin derivatives (Section 7.2) started phase 1 clinical trials in 2020 and 2021, respectively [134]. Interestingly, alongside the clinical trials of new antibacterials, the older ones are used in innovative combinations. For instance, ciprofloxacin, a member of fluoroquinolones class, is under clinical trial as a component of a smart gel in combination with chitosan-coated polymer nanoparticles, for use as in situ gel to be injected into an infected root canal [135].

**Figure 13:**
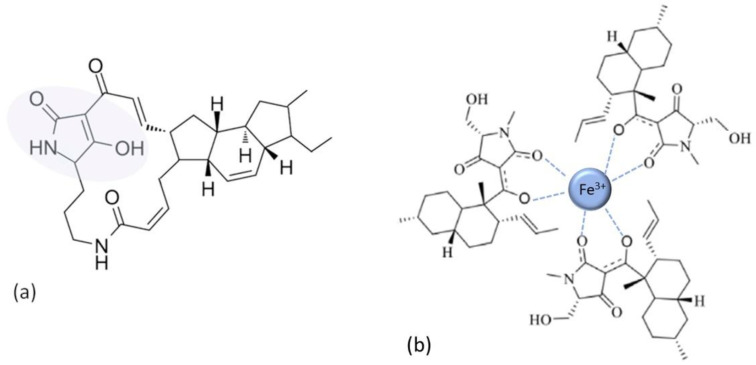
(**a**) Ikarugamycin, a member of the tetramate class and the polycyclic tetramate macrolactams (PTM) subgroup. Tetramate moiety is emphasized in the shaded area. (**b**) An example of the tetramate chelate, comprising three tetramate (equisetin) molecules as bidentate ligands chelating Fe^3+^ cation. Inspired by ref. [72].

The chelating strength of the substances is being used to predict their biological activity on bacterial cells [136,137]. The search for alternative substances to small molecule antimicrobials has identified chelation therapy as one of the main approaches, with the potential for the treatment and prevention of antibiotic-resistant infections [138] and for the design of advanced antimicrobial approaches [139]. The example of such substances are tetramates, the derivatives of tetramic acid (Figure 13; tetramate moiety [140] is emphasized in Figure 13a). This class emerged recently as potential new antibiotics as a consequence of the search for antimicrobials mimicking natural products with chelating properties.

Along with, e.g., polyphenols and quinones, the search yielded 3-acyltetramic and tetronic acids (and their derivatives), which demonstrated activities against multidrug-resistant bacteria [72]. Their antibacterial actions are shown to be directly correlated to their chelating ability [73]. As opposed to simpler fungal tetramates, bacteria tend to produce a polycyclic tetramate macrolactams (PTM), which are especially promising (Figure 13a) [74]. The proposed antibiotic MOA includes the chelation of Fe^3+^ cation (Figure 13b), followed by a reduction of Fe^3+^ to Fe^2+^ when a chelate diffuses into a high pH area, which in turn triggers Fenton reactions, i.e., oxidative attack by the production of deleterious hydroxyl radicals. Interestingly, due to the connection to Fenton chemistry and the fact that PTMs can be found in sugar beet rhizosphere soil, the tetramates have the potential to provide a solution for biocontrol against crop infections [72].

## 11. Conclusions

This review presents a survey of MOAs of the majority of antibacterial classes from the perspective of chelation. The chelating ability of the drug is crucial for most MOAs, with a staggering spectrum of roles it can assume. For some drugs, such as century-old nitroxoline and just-discovered tetramates, it determines their overall bioactivity, and provides a platform for the expansion of activities beyond antibacterial. For others, such as cefiderocol and vancomycin, it provides the means to improve the basic structure in order to fight the ever-increasing bacterial resistance. For some that do not entail the chelation in their MOAs, again, it emerges as the drug target (polymyxin) or as a means of help from adjuvant substances (e.g., zinc chelators for carbapanem class). Furthermore, it provides (unfortunately) a successful mechanism of bacterial resistance, illustrated by metallo-β-lactamase actions. Hence, understanding and exploiting the chelation processes seems to be a valuable subject for future research. As emphasized by Johnstone and Nolan [23], the emergence of antibiotic-resistant bacteria in recent years has highlighted more than ever the importance of understanding the fundamental chemistry underlying bacterial life.

## Data Availability

Not applicable.
